# Exploring emotions in Bach chorales: a multi-modal perceptual and data-driven study

**DOI:** 10.1098/rsos.230574

**Published:** 2023-12-20

**Authors:** Emilia Parada-Cabaleiro, Anton Batliner, Marcel Zentner, Markus Schedl

**Affiliations:** ^1^ Institute of Computational Perception, Johannes Kepler University Linz, Linz, Austria; ^2^ Human-Centered AI Group, AI Laboratory, Linz Institute of Technology (LIT), Linz, Austria; ^3^ Department of Music Pedagogy, Nuremberg University of Music, Nuremberg, Germany; ^4^ Chair of Embedded Intelligence for Health Care and Wellbeing, University of Augsburg, Augsburg, Germany; ^5^ Department of Psychology, University of Innsbruck, Innsbruck, Austria

**Keywords:** multi-modality, emotion, symbolics, acoustics, linguistics, sacred music

## Abstract

The relationship between music and emotion has been addressed within several disciplines, from more historico-philosophical and anthropological ones, such as musicology and ethnomusicology, to others that are traditionally more empirical and technological, such as psychology and computer science. Yet, understanding the link between music and emotion is limited by the scarce interconnections between these disciplines. Trying to narrow this gap, this data-driven exploratory study aims at assessing the relationship between linguistic, symbolic and acoustic features—extracted from lyrics, music notation and audio recordings—and perception of emotion. Employing a listening experiment, statistical analysis and unsupervised machine learning, we investigate how a data-driven multi-modal approach can be used to explore the emotions conveyed by eight Bach chorales. Through a feature selection strategy based on a set of more than 300 Bach chorales and a transdisciplinary methodology integrating approaches from psychology, musicology and computer science, we aim to initiate an efficient dialogue between disciplines, able to promote a more integrative and holistic understanding of emotions in music.

## 1. Introduction

**Figure 1.**
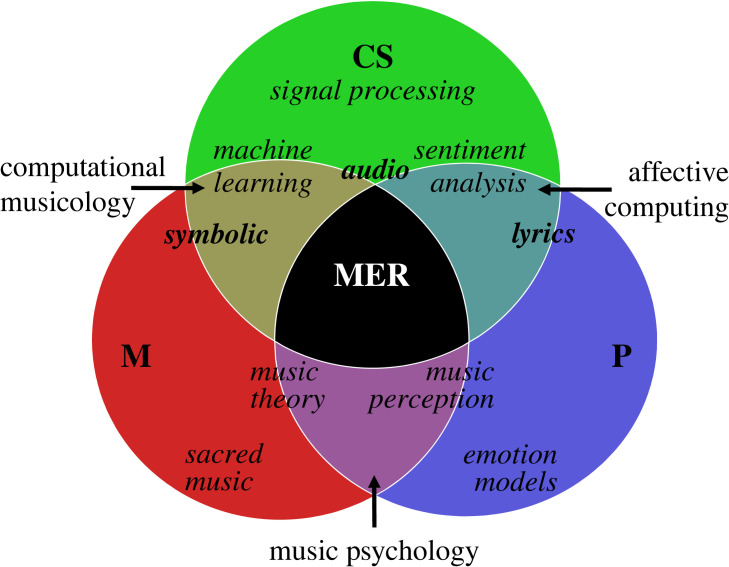
Representation of the three main disciplines studying music and emotion: musicology (M), psychology (P), computer science (CS); the sub-fields at their intersection: music psychology (between M and P), affective computing (between P and CS), computational musicology (between CS and M). Relevant components within and across these disciplines and sub-fields are indicated: the musical representations typically investigated (audio, symbolic, lyrics) are highlighted in bold; music emotion recognition (MER) is indicated in the centre of the diagram.

Associations between music and emotion are investigated in a variety of disciplines, from psychology [1] to computer science [2] and the humanities [3]. Nevertheless, exchanges between these disciplines are often restricted, thereby hindering the evolution of emerging disciplines such as digital humanities [4]. Correlations have been found between specific emotions and particular musical parameters [5]. Yet, it is still not fully understood whether concrete musical properties can coherently and systematically convey emotions when extracted from different musical sources. This is in part owing to the lack of a *transdisciplinary methodology*, that is, a shared conceptual framework aimed to solve a common problem [6]. The present study is based on the premise that a holistic understanding of musical emotions would be encouraged by the application of a transdisciplinary approach. This approach, jointly derived from methods from psychology, musicology and computer science, is built upon interdisciplinary domains of knowledge, including perception, music theory, machine learning (ML) and sentiment analysis. In figure 1, a diagram is shown displaying the essential pillars in the development of the transdisciplinary methodology used in this study to investigate musical emotions.

Research on musical emotions is typically centred around the debate on whether music is capable of just expressing [7] or also inducing [8] affects. Beyond this debate, emotion research in a variety of domains, such as neuro-science [9] and affective computing [10], seems to agree that emotions are subjective experiences. Thus, especially in this domain of knowledge, no *ground truth* can be unequivocally determined, but a *gold standard* might be aimed at, for instance, with the help of perceptual evaluation (annotations). Concerning emotional responses to music, the inherent subjectivity of emotions is complicated even further by the presence of underlying mechanisms, e.g. visual imagery, one of several mechanisms, beyond cognitive appraisal, through which music listening may induce emotions [11]. In this context, developing a transdisciplinary methodology might be encouraged by choosing a musical repertoire with inherent emotional connotations, for instance, works in the realm of sacred music. On the one side, religious repertoire often expresses the spiritual content encoded in the liturgical text [12]. On the other side, it normally contains the singing voice as a central component—worldwide across religious traditions [13]—which enhances its emotional connotations [14]. This is also supported by evidence of acoustic correlations between emotional expression in speech and singing [15]. Since from a vocal sacred work, specific musical features aimed to highlight particular spiritual and emotional concepts can be retrieved [16], this type of repertoire is particularly suitable to investigate musical emotions, as shown by works within ethnomusicology [17] and musicology [16]. Yet, within music psychology and music information retrieval, emotions in sacred music have not been addressed so far.

Datasets containing both emotion annotations and the musical sources, from which a variety of features can be extracted, can typically be found in the context of music emotion recognition (MER). Nevertheless, most of the currently available datasets within MER display a clear bias towards audio sources [18].^1^ By that, audio signal processing dominates MER research [2,19,20]. However, it has been shown that other sources, such as lyrics, are also an important source to be taken into account when assessing musical emotions: by extracting emotional information from song-lyrics through methods from natural language processing (NLP), notably sentiment analysis [21], the understanding of emotions in music has been encouraged. Studies assessing emotions conveyed by lyrics have also been conducted in the realm of music psychology [22,23]. Similarly, a systematic evaluation of how lyrical emotions are expressed through musical, symbolically encoded features has also been attempted [24]. However, to the best of our knowledge, the study by Sun & Cuthbert [24] is the only one using *codified scores* as a source to investigate musical emotions.^2^ This is not trivial because even though the use of specific composition strategies (accessible from musical scores) for conveying particular affects in Western music is well established [7], using codified scores as a source for MER remains almost unexplored. The cited works demonstrate the potential of using symbolic sources (both lyrics and codified music) to study musical emotions. Yet, since multi-modal datasets, containing not only audio but also symbolic representation, are rather the exception than the rule [25], research based on lyrics, codified scores and recordings is carried out independently from each other. Thus, we cannot compare conclusions drawn from studies employing different musical modalities.

As a first attempt to redress this lacuna, we performed a data-driven exploratory study, presented as a proof-of-concept of the underlying transdisciplinary methodology, which aims at investigating the relationship between multi-modal musical attributes and perception of emotions in eight Bach chorales. In order to increase the generalizability of our results, even if the research questions (RQs) examine eight chorales, we selected machine-based relevant features from more than 300 Bach chorales (Bach300+): for symbolic the whole set of Bach chorales excluding the eight evaluated, i.e. 362; for the acoustic, the 300 chorales that were available in a comparable recording set-up—again, these do not contain the eight evaluated. Then, we performed an in-depth perception study where ratings of perceived emotions by 26 participants are assessed with both dimensional and domain-specific categorical models of emotion. Subsequently, we evaluate to which extent perceived emotions can be explained with features extracted from three modalities: lyrics, codified scores and audio recordings. Moreover, to promote further research on the topic, we make the resources necessary to reproduce the outcomes of our exploratory study freely available.^3^ Our investigation, summarized in table 1, assesses the following three RQs:

— RQ1 investigates to which extent the perception of emotion in the selected repertoire can be related to the musical properties identifiable by analysing the musical score. This is the type of assessment typically done in musicology and music psychology; cf. user-based study in table 1 (§§2.1 and 3.1);
— RQ2 analyses whether relationships between perception of emotion and machine-based features automatically extracted from the investigated repertoire do exist. For this, three types of features (linguistic, symbolic and acoustic) are automatically computed from three representations (lyrics, codified scores and recordings); cf. data-driven study in table 1 (§§2.2 and 3.2); and
— RQ3 examines potential connections between the emotional characterizations of the music as obtained from perception and as generated from unsupervised ML techniques based on multi-modal features. Thus, RQ3 goes a step beyond the perceptual experiment (investigated in RQ1) and the data-driven one (investigated in RQ2), by investigating multi-modal relationships; cf. multi-modal study in table 1 (§4).

**Table 1.** Summary of experiments.

study	features (source)	experiments	analysis
user-based (RQ1)	none (recordings)	2 (dimensional and categorical model)	interpretation of listeners’ ratings (perception of the recordings) according to analytic principles from music theory (assessed in the scores)
data-driven (RQ2)	linguistic (lyrics)	2 (dimensional and categorical model)	interpretation of the lyrics’ emotional mapping (using linguistic features from sentiment analysis) according to listeners’ ratings of emotion
	symbolic (scores)	1 (overall evaluation of both models)	interpretation of correlations (based on symbolic features extracted from the encoded music) across chorales according to listeners’ ratings of emotion
	acoustic (recordings)	1 (overall evaluation of both models)	interpretation of correlations (based on acoustic features extracted from the audio files) across chorales according to listeners’ ratings of emotion
multi-modal (RQ3)	linguistic (lyrics)	1 (overall evaluation of both models)	interpretation of the clustering results considering features extracted from single/multiple modalities, according to listeners’ ratings of emotion
	symbolic (scores)		
	acoustic (recordings)		

## 2. Methods

### 2.1. User-based study

#### 2.1.1. Dataset

To the best of our knowledge, the only dataset with emotional annotations containing the three chosen modalities (lyrics, codified scores and audio recordings) is the one by Panda *et al.* [25], which—having a focus on commercial genres—does not include sacred music. Given this limitation, for the present study, we chose the Bach10 dataset [26]. Although Bach10 was developed for tasks such as audio-score alignment or source separation (i.e. not for MER), it is suitable for our purposes since it contains both codified music notation (in form of MIDI files) and audio recordings of 10 Bach chorales, that is, sacred music. The dataset is characterized by two additional important features: (i) it consists of recordings with four different instruments, violin, clarinet, saxophone and bassoon, playing the Canto, Alto, Tenor and Bass parts, respectively;^4^ and (ii) in the performances, ‘correctness’ is prioritized over expressiveness. Thus, the recordings sound to some extent ‘mechanical’: in order to accurately perform, for instance, the rhythms, expressive mechanisms such as *rubato*—an elastic and flexible conception of the tempo [27]—were minimized. Since it has been shown that voice expressiveness can convey some emotions significantly better than other instruments [28], to assess how scoring (i.e. the instrumentation) and expressiveness (i.e. the performance) might cause differences in the perceived emotions, besides Bach10 we also assessed recordings of the exact same chorales performed by a professional choir.^5^ From now on, we will refer to the recordings from the dataset as ‘Bach10’ and to those by the choir as ‘Kantorei’. Using the Bach10 dataset gives us the unique opportunity to assess the role of scoring and performance while using real performances. Note that this cannot be done by directly playing the MIDI synthesized files in the listening test instead of the Bach10 recordings: while the Bach10 recordings sound not so expressive as the Kantorei recordings, they still sound real. This is not trivial; in such a scenario, understanding whether eventual differences in perception might be owing to the artificial audio (i.e. MIDI) or to the differences in instrumentation and performance, would have not been possible.

Eight chorales were selected for the study: four in major key, four in minor key. Note that this is an exploratory study with a limited dataset. We do not aim at a shallow large-scale corroboration of specific hypotheses but at a thorough and detailed investigation of all the components that contribute to our understanding of the intricate interdependence of music, lyrics and performance. In table 2, a summary of these chorales including the listeners’ annotations for the Kantorei recordings and cadences of each verse are given. When available, the cadences were extracted from existing harmonic analyses^6^; otherwise, they were performed by the authors. The cadences are indicated and will be referred to in the discussion of the results as an indicator of harmonic stability. Each chorale is expected to represent a relatively cohesive emotion; yet, in order to evaluate and compare the chorales as unique musical entities, they are not segmented into their individual verses; i.e. both listeners’ perception and features are extracted at the chorale level. In table 2, ♭VII is used to indicate the subtonic chord for major tonalities. By contrast, since in the minor ones the subtonic is part of the natural scale, VII is indicated instead—a chord typically used as dominant of the relative major (V/III), as described by Kostka *et al.* [29].

**Table 2.** For each chorale: identifier (a word from the first verse for referring to each chorale in the text), ID from the Bach10 dataset, beginning of first verse, tonalities, category and percentage of the most frequently chosen emotion (perceived from the ‘real’ performances), musical cadences of each verse (upper case indicates major chords, lowercase minor ones), sample length in seconds (Kantorei before, Bach10 after the slash), as well as mean perceived valence (V) and arousal (A) for the Kantorei recordings.

choral	ID	beginning of verse	tonality	emotion	%	cadences	s	V	A
Solls	B01	*Solls ja so seyn …*	C major	power	42.3	V; I; V; V; V; I	39/26	0.65	2.08
Schlafen	B02	*Wir wachen oder schlafen …*	A minor	power/transcen.	34.6	‖:III; I:‖ V; VII; I	83/40	0.46	2.27
Tag	B03	*Christe, der du bist Tag …*	G minor	transcen.	73.1	i; VII; i; I	31/25	0.15	2.50
Beistand	B04	*Christe, du Beistand …*	D minor	transcen.	61.5	V; v; III; VII; v; iv; I	78/41	0.00	2.04
Nacht	B05	*Die Nacht ist kommen …*	G major	transcen.	53.9	I; ii; V; I; ♭VII; V; I	78/35	0.46	2.23
Sonn	B06	*Die Sonn hat sich …*	D minor	transcen.	46.2	V; III; III; I	42/36	−0.04	2.15
Thron	B08	*Für deinen Thron …*	D major	transcen.	53.9	I; V; vi; I	37/33	0.38	1.81
Lieb	B10	*Du süße Lieb’ …*	A major	power/transcen.	30.8	I; I; vi; IV; V; I	52/37	1.19	2.65

Finally, the English translations of the lyrics were taken from the *Chorales* section of the Bach Cantatas website.^7^ Unlike automatically translated lyrics, this source, containing human-curated translations, was chosen as a more reliable alternative. The quality of the translations was additionally approved by a native German speaker. We refer to the verse put in music by Bach, that is, the one considered in the Kantorei recordings, which for some chorales is not the first, thus differing from the indicator given by Bach10.^8^ The translations of the lyrics are only used to automatically extract linguistic features, as the existing methods for sentiment analysis, such as embeddings and emotion lexica, have been developed in English. The listeners were expected to understand the lyrics’ meaning, since all were native (or in a few exceptions fluent) German speakers. Yet, we did not provide any lyrics (neither the original nor the translations) or referred to them in any way during the listening experiment, as our goal was to assess both musical excerpts (i.e. Kantorei: containing lyrics; Bach10: without lyrics) with the very same procedure.

#### 2.1.2. Listening experiment

A total of 44 students (three female, 41 male) from the curricula in Computer Science and Artificial Intelligence at the Johannes Kepler University Linz (Austria) participated in the study. The procedures used adhere to the tenets of the Declaration of Helsinki; written consent was obtained from the participants to use their anonymous responses for research purposes. Owing to the highly imbalanced gender distribution, the three female students were excluded, in order to be able to study a homogeneous cohort. Furthermore, to avoid familiarity and disliking as confounding factors, participants were also requested to indicate yes/no about their familiarity with and liking of the evaluated music. Only students who indicated to be familiar and to like the music were included in the study, resulting in the responses of 26 male students to be evaluated. All the selected students were Austrian except two, who, however, were both fluent in German.

In the listening experiments, we assess perceived emotions, that is, the emotions cognitively ascribed from a listener perspective *to the music itself*, as the ones described by Kivy [7].^9^ Concerning the theoretical framework taken as reference, most of the research in music and emotion is carried out employing two main models of emotion: the categorical, which identifies emotions with concrete categories such as the ones described by Ekman [30]; and the dimensional, which identifies emotions within a continuous hyperplane delimited by emotional dimensions, such as arousal and valence, as described by Russell [31]. Attempts to understand which of these two models is more suitable to investigate emotions in music have been carried out [32,33]; recent research even questions the theoretical foundations of valence and arousal as fundamental components of subjective experiences [34]. There exist fundamental differences between universal emotions and emotions evoked by music—such as the role played by underlying mechanisms [11]. Therefore, it was necessary to develop models specifically tailored to investigate emotions in music; examples are the one by Zentner *et al.* [35] and the one by Hevner [36]. For a comprehensive review of methods to measure emotions in music, we refer to Zentner & Eerola [37].

In order to enable the interpretation of our results according to previous works, ratings of perceived emotion were obtained using (i) a scale based on the dimensional circumplex model by Russell [31] that represents emotions within the two-dimensional space of arousal and valence that is often used in studies on music and emotion, and (ii) a domain-specific categorical model, the Geneva Emotion Music Scale (GEMS), which is derived from a model that was specifically devised to account for musically evoked emotions [35]. The model is hierarchical and consists of three superordinate emotion factors (sublimity, vitality and unease) and nine primary emotion factors. Although GEMS was primarily devised to assess felt emotion, the nine-factorial model was also found to account rather well for perceived emotions; see appendix C in [35] and [38]. In the present study, we use a slightly modified 10-factorial version of GEMS, which has already been used for the purpose of describing perceived (cognitively ascribed to the music) in addition to felt (induced in the listener) emotions; see study 2 in [35]. The factors within the three highest levels of abstraction (sublimity, vitality and unease) are: amazement, sadness, sensuality, transcendence, tenderness and tranquility (sublimity); activation, joy and power (vitality); dysphoria (unease).

The listening experiment was performed with headphones through a web-based interface. The participants could listen to the randomized recordings more than once but were encouraged to give spontaneous responses. Every participant rated every stimulus, which had to be listened to in its entirety at least once before being able to insert the perceived emotion. The students were instructed to indicate the emotion perceived for each stimulus through two rating scales (to assess arousal and valence) and a multiple-choice categorical test; see details below. Note that all the participants were familiar with the emotional models as well as the assessment methods used in the experiment, as they were all attending the course ‘Affective Computing’, of which the listening experiment was part. After rating all the stimuli, the participants completed a short questionnaire indicating their familiarity with and liking of the listened repertoire (binary rating: yes/no) as well as their gender (female, male or other). Although the listening test was expected to last around 30 min, this time varied across participants depending on their individual differences and interest in the task. Note that this type of perceptual experiment differs with respect to the annotation procedures based on crowdsourcing, where different samples are annotated by different listeners, and confounding factors such as listeners’ familiarity and liking are not considered. We would like to emphasize that performing a perceptual study of this kind on the 370 Bach’s chorales would not be possible owing to the human resources needed. Even if the listeners were expected to annotate only one type of recording, they would need at least 15 min per 10 chorales, which means that 9 h and 25 min would be needed (without considering the breaks) by each of the approximately 40 participants to annotate the whole dataset. Moreover, such a large-scaled experiment would imply additional problems such as dropouts and intralabeller consistency, and/or the employment of different labellers for different parts of the data.

For the dimensional assessment, the valence and arousal dimensions were assessed separately with two rating scales of five levels: from 0 to 4 for arousal and from −2 to 2 for valence. Note that arousal starts low and can increase while valence can be either negative (below 0) or positive (above 0); thus, it is semantically more adequate to employ two different scales. For the categorical assessment, the 10-factorial GEMS were presented in a multiple-choice format. Along each factor, the respective adjective markers (cf. table 1 in [35]) were provided in order to illustrate the factors’ meaning. In the dimensional assessment, the participants could select only one value for each dimension; in the categorical assessment, they could choose only one out of the 10 emotional factors. The ratings were static annotations performed for the whole sample. This method was preferred over continuous annotations owing to the short length of some samples; cf. sample length in seconds (s) in table 2. Note that at least 15 s—more than half for some samples—might be needed as the *orientation time* [39], that is, the initial period in continuous annotations during which the collected ratings are unreliable and should be discarded.

### 2.2. Data-driven study

#### 2.2.1. Linguistic features

The lyrics of each chorale were automatically mapped onto the emotional dimensions and categories using emotion lexica and word embeddings. Emotion lexica are lists of words rated in terms of their emotional value, while word embeddings are a representation of words as real-valued vectors in a predefined multi-dimensional space where the similarity of words is represented by the vectors’ proximity. Note that in religious texts, the use of explicit words in order to clearly convey particular emotions is much more evident than in the type of text typically used in NLP for sentiment analysis, such as product or film reviews. The reason is that in religious music, the lyrics also have a pedagogical purpose, namely transmitting the inherent emotional tone [40]. Indeed, besides musical symbols, Bach used to exploit the actual sung words as a strategy to convey meanings as well [41]. Thus, the emotional value of a religious text is often related to the emotional value of the specific words within the text, which makes emotion lexica and word embeddings promising computational resources.^10^ We used the *emotion word embeddings* (EWE) by Agrawal & Papagelis [42], as well as three lexica: The extended Affective Norms for English Words (ANEW; [43]); National Research Council (NRC) Canada Lexicon [44] and Valence Aware Dictionary for Sentiment Reasoning (VADER; [45]). As already mentioned, since both the embeddings and the lexica are in English, the translated lyrics were used for the data-driven study instead of the original in German. For obvious reasons, English archaic forms such as *drivest* or *seemeth* were replaced by modern forms. Although the used embeddings (EWE) are emotion-enriched word representations where the affective component is prioritized over the semantic one, i.e. emotionally similar words are projected into neighbouring spaces even if their contexts are dissimilar, it is important to note that the text data used to train such embeddings comes from fairy tales, blogs, experiences and tweets [42]. Owing to this, these sources are expected to be far away from the chorales’ lyrics used in our study, whose poetic and metaphorical nature might not be completely captured by the used representations. Still, as the word embeddings are weighted according to emotion lexica, which are agnostically (i.e. without context) rated in emotional terms by humans, we could assume a sort of ‘objective’—albeit not stylistic—validity in the used resource.

##### 2.2.1.1. Dimensional mapping

Each word *i* in the lyrics was mapped onto a four-dimensional vector *i* = (*v*, *a*, *d*, *p*), where *v*, *a* and *d* stand for the valence, arousal and dominance scores from ANEW and *p* stands for polarity, that is, the compound measure from the NLTK’s VADER module^11^; words missing in ANEW were mapped onto NRC. To enable comparability with the perceptual results, the elements of the four-dimensional vectors were linearly scaled according to the values used in the listening experiment: the same scale as for arousal was adopted for dominance; the same as for valence was adopted for polarity. Subsequently, as common in lexicon-based approaches [46], the weighted arithmetic mean (*μ*) and the standard deviation (*σ*) across the four-dimensional vectors representing every word in each chorale *C* = (*i*_1_, *i*_2_, …, *i*_*n*_) were computed. Thus, the lyrics of a given chorale are represented by an eight-dimensional vector *l* = (*μ*_*C*_, *σ*_*C*_).

##### 2.2.1.2. Geneva Emotion Music Scale mapping

A mapping between the lyrics and the GEMS factors was performed by computing the cosine similarity between the EWE [42] of each term in the lyrics and of the adjective markers (representing each factor). Before performing the mapping, the text was pre-processed according to standard procedures, including the tokenization of the lyrics into individual words, stop-words removal and lemmatization, i.e. converting a word into its root; this was preferred over stemming, as it is a less invasive solution. To enhance the distances between factors in the embeddings, we weighted them according to the emotion lexica. As a first step, each word of the embeddings was mapped onto a four-dimensional vector *i* = (*v*, *a*, *d*, *p*) following the procedure described in the dimensional mapping. Subsequently, the 300-dimensional pre-trained vectors from the embeddings *E* representing each word were multiplied by each element of *i* (corresponding to the same word) and concatenated, resulting in 1200-dimensional vectors. In short, each word vector is defined as *W* = (*E*_1_*v*, *E*_1_*a*, *E*_1_*d*, *E*_1_*p*, …, *E*_300_*v*, *E*_300_*a*, *E*_300_*d*, *E*_300_*p*). Finally, to enable the mapping of words from the embeddings not contained in the lexica in the weighted space, a transformation matrix was generated by computing the linear projection of the weighted subset and the part of the original pre-trained vectors containing the same words. The vectors representing the remaining words in the weighted space were computed by multiplying the 300-dimensional pre-trained vectors by the transformation matrix. The number of adjective markers varies across GEMS factors; to guard against a bias resulting from this unequal distribution, we selected three adjectives per factor, prioritizing those appearing in the embeddings, i.e. selecting the first from the list appearing in the embeddings.^12^ As many words in the chorales had no emotional connotations, their similarity was close to 0; thus, to counterbalance the noise caused by the non-emotional terms, all the words with a mean similarity greater than or equal to 0.3 across the three adjective markers were considered as if they appeared twice in the lyrics. The naturally appearing threshold of 0.3 was chosen based on visual inspection, i.e. by plotting the mean cosine similarities between chorales and factors. Subsequently, the arithmetic mean across the similarities between every chorale’s word and the adjectives was computed: defining the embeddings of the *f*th GEMS factor as *G*_*f*_ = (*G*_*f*1_, *G*_*f*2_, *G*_*f*3_) for the three adjectives and the number of words *K*, the mean similarity for each factor is defined as $M_{f}=1/KJ\sum_{k}\sum_{j}\;\mathrm{csim}\;(W_{k},G_{\;fj})$, with the cosine similarity csim.

#### 2.2.2. Symbolic features

From the MIDI files, we extracted the default feature set of jSymbolic 2.2 [47], which encompasses a variety of descriptors related to pitch (both absolute and pitch classes), melody (both melodic and horizontal intervals), texture (related to the interaction between the independent voices), rhythm (related to notes’ attacks and durations), vertical intervals (including chords and the harmonic movement) and dynamics (related to notes’ expressive component, including intensity and articulation). These descriptors are suitable to automatically capture emotional content from MIDI, as described by Panda *et al.* [48] as *novel audio features*. From the mentioned parameters, statistics (e.g. frequencies, range or mean) are computed at score level, considering each chorale as a whole. After excluding features that were not meaningful in the evaluated repertoire, e.g. micro-tones, a total of 188 features remained. In order to identify a meaningful subset of features, representative of the investigated repertoire, the feature selection strategy was applied on the whole set of chorales excluding the eight chorales which will later on be evaluated, i.e. the 362 chorales available in the github repository by Craig Sapp ‘bach-370-chorales’.^13^

**Figure 2.**
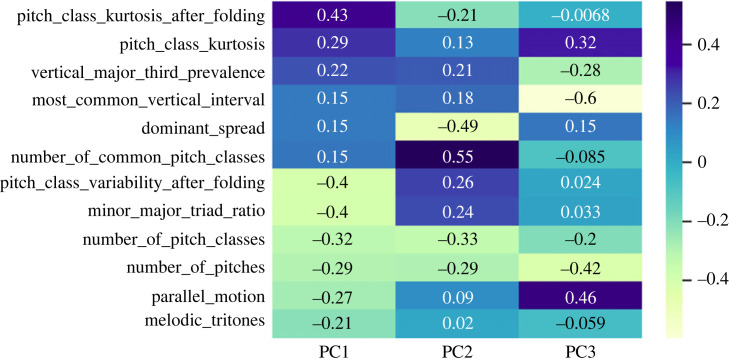
Component loadings between the 12 selected symbolic features and the three first principal components computed on the whole Bach300+ symbolic dataset, i.e. 362 chorales.

As a first step, in order to enable comparability across the feature vectors, the chorales were automatically transposed to C with the python library music21. Subsequently, features were normalized and those with very low variance across samples were removed. Finally, as a method for dimensionality reduction, principal component analysis was used, by this selecting the features with a correlation with the first principal component (PC) > |0.1|. After excluding the least correlated features (this implied a drop from 43 to 12 features), the mean explained variance increased from 2.3% to 8.3% while the variance explained from the first PC increased from 11% to 35%. A total of 12 relevant features (shown in figure 2) were identified through the feature selection strategy. Although interpreting the type of information encoded by each PC might be difficult, we could hypothesize that the first PC, positively correlated with features related to the pitch class, vertical intervals as well as dominant spread, might be able to capture the harmonic information. At the same time, the coefficients of this PC are negatively correlated with features related to melodic intervals and type of motion, thus disregarding information representing the melodic contour.

#### 2.2.3. Acoustic features

From both types of recordings, we extracted the 494 first level statistical functionals, computed from the 26 low level descriptors (LLDs) of the emobase feature set, with the openSMILE toolkit [49]. This feature set was chosen since it has been developed to capture emotional content from audio. Apart from its successful use in the music domain in general [50,51], emobase is specially tailored to process voice, as shown by its specific vocal features. Thus, owing to the prominent role of the singing voice in the investigated repertoire, emobase was considered the most suited feature set. Note that the typical features relevant for MER directly computed from the audio signal [20], for instance those related to dynamics and timbre, are included in emobase, as openSMILE is a standardized toolkit in affective computing. The emobase feature set encompasses a variety of LLDs related to zero-crossing rate (frame-based of the time signal), intensity (energy), loudness (normalized intensity raised to a power of 0.3), probability of voicing (computed via an autocorrelation function and cepstrum based method), line spectral frequencies (the eight line spectral pair frequencies computed from eight linear predictive coding coefficients), pitch (F0, i.e. fundamental frequency computed from the Cepstrum) and envelope (smoothed fundamental frequency contour). From these, statistical functionals such as maximum, minimum or range, are computed. In order to identify a meaningful subset of descriptors, representative of the investigated repertoire, the same selection strategy described for the symbolic features was applied, this time on a set of 305 chorales. The corresponding audio was retrieved from Youtube, provided by the classical music label *Brilliant Classics* and performed by the *Chamber Choir of Europe* with Nicol Matt as conductor. Although gathering consistent recordings for the 370 chorales was not possible (i.e. recordings performed by the same choir), we believe that the collected amount of 305 audio recordings suffices to guarantee a reliable feature selection able to capture the unique characteristics of the investigated repertoire.

**Figure 3.**
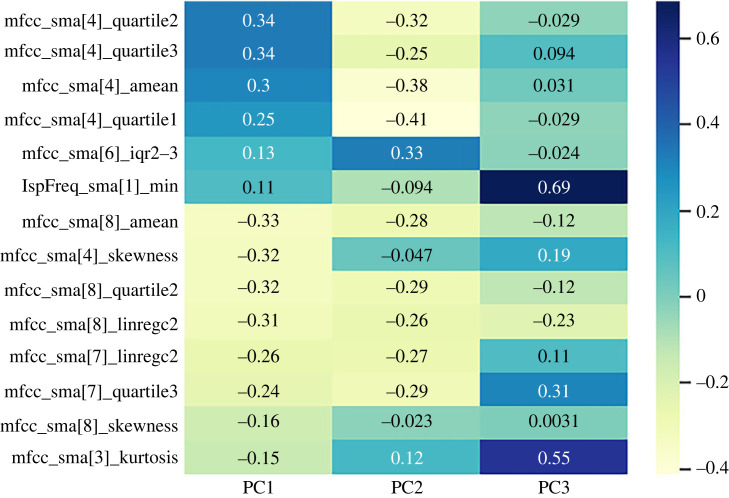
Component loadings between the 14 selected acoustic features and the three first principal components computed on the whole Bach300+ audio dataset, i.e. 300 Bach’s chorales.

As for the symbolic data, before performing the feature selection, the eight chorales that are subsequently evaluated were excluded from the Bach300+ audio dataset. Since only five out of the eight chorales were present in the whole audio dataset, 300 chorales were taken into account for the audio feature selection. After carrying out the feature selection, excluding the least correlated features yielded an increase in the mean explained variance from 4% (with 24 features) to 7.1% (with 14 features), while the variance explained from the first PC increased from 24% to 42%. A total of 14 relevant features (shown in figure 3) were identified through the feature selection strategy. If interpreting the component loadings was challenging for the symbolic features, this becomes even more difficult for the acoustic ones. As we can see, all but one of the selected features are Mel-frequency cepstral coefficients (MFCC), i.e. features describing the spectral characteristics of the sound. For the first PC, most impactful are the fourth coefficients, encoding mainly slow variations of the spectrum, while higher coefficients (seventh and eighth) are negatively correlated. This might be interpreted as PC1 encoding information related to lower frequencies.

## 3. Results

### 3.1. User-based study

In this section, we address RQ1: *to which extent can the perception of emotion in the selected chorales be related to their musical properties?* We will first examine the listeners’ responses and their reliability for both types of recordings (Kantorei and Bach10), separately for the dimensional and the categorical assessment (cf. §§3.1.1 and 3.1.2, respectively). An in-depth discussion of participants’ affective responses to the music in relation to its musicological properties is then provided in §3.1.3.

Owing to the differences in perception for each type of recording, reliability was assessed considering each recording type and emotional dimension individually. For the dimensional assessment, the intraclass correlation coefficient (ICC) with a *two-way random-effects* model and *agreement* as definition parameter was computed. As we want to employ the perceived mean value across raters for each emotional dimension as a gold standard, the *average* option was chosen as the suitable type for the ICC [52]. For the categorical assessment, Gwet’s agreement coefficient (AC_1_) [53] was computed on the three highest levels of abstraction. Gwet’s AC_1_ was chosen as a more stable alternative in comparison to other inter-rater ACs such as the kappa statistic, which heavily depends on the experimental set-up [54]: more categories might yield a lower kappa even with identical raters, which has been identified as ‘the paradoxes of kappa’ [55].

After examining the reliability of the perceptual ratings, the results will be interpreted according to principles of music theory, such as mode or harmonic stability. In order to analyse the relationship between dimensional and the categorical ratings, results of a point biserial correlation carried out between each dimension and the pairwise (dichotomously encoded as ‘1’ and ‘0’) most frequently chosen emotional categories will be also discussed (cf. §3.1.3).

#### 3.1.1. Dimensional assessment

The reliability of the dimensional ratings was comparable for both types of recordings: the perception of the Kantorei and Bach10 recordings yielded an ICC of 0.81 and 0.85 for valence, and of 0.54 and 0.40 for arousal, respectively. By comparing listeners’ dimensional ratings for both types of recordings given in table 3, we observe that valence spans over a larger range of values than arousal. For the perception of **valence**, the range between the minimum and maximum ratings (italics values in table 3) is above 1 for both types of recordings: for Kantorei, −0.04 ≤ valence ≤1.19, valence range = [1.19 − ( − 0.04)] = **1.23** ; for Bach10, −0.88 ≤ valence ≤0.54, valence range = [0.54 − ( − 0.88)] = **1.42**. By contrast, for **arousal** the range is below 1 for both types of recordings: for Kantorei, 1.81 ≤ valence ≤2.65, valence range = [2.65 − 1.81] = **0.84**; for Bach10, 1.38 ≤ valence ≤2.00, valence range = [2.00 − 1.38] = **0.62**. This means that, from a listener perspective, differences between the chorales’ valence are displayed. Some are perceived as positive (cf. *μ* = 1.19 for Lieb in table 3; this agreement among raters is shown by the low *σ* = 0.57).^14^ Others are perceived as negative (cf. *μ* = −0.88 and *σ* = 0.86 for Beistand in table 3). By contrast, no clear arousal differences are observed. In both dimensions, the Kantorei recordings are generally perceived with higher values than the Bach10, that is, the chorales are perceived as more positive and having a more intense arousal. The average *μ* across chorales is, for Kantorei versus Bach10, 0.41 versus −0.11 (valence), and 2.22 versus 1.80 (arousal). Note that *p*-values adjusted for multiple testing yield a significant difference in both dimensions only for the chorale Lieb.

**Table 3.** Average ratings and standard deviation (*μ* ± *σ*) for the dimensional perception (valence and arousal) across listeners for each chorale in the two recording types (Kantorei and Bach10). (The extreme values for each dimension and recording type, i.e. highest and lowest per row, are highlighted in italics.)

		chorale							
		Solls	Schlafen	Tag	Beistand	Nacht	Sonn	Thron	Lieb
valence	Kantorei	0.65 ± 0.94	0.46 ± 0.90	0.15 ± 0.83	0.00 ± 0.94	0.46 ± 1.03	−*0.04* ± 1.00	0.38 ± 0.90	*1.19* ± 0.57
	Bach10	0.19 ± 0.90	*0.54* ± 1.07	−0.62 ± 0.90	−*0.88* ± 0.86	−0.04 ± 1.15	−0.54 ± 1.17	0.00 ± 1.17	0.46 ± 1.10
arousal	Kantorei	2.08 ± 1.20	2.27 ± 1.04	2.50 ± 0.95	2.04 ± 0.96	2.23 ± 1.18	2.15 ± 1.01	*1.81* ± 0.98	*2.65* ± 0.80
	Bach10	1.65 ± 0.85	*2.00* ± 0.75	1.81 ± 0.94	1.92 ± 1.93	*1.38* ± 0.70	1.81 ± 0.90	1.88 ± 0.95	1.92 ± 0.89

#### 3.1.2. Geneva Emotion Music Scale assessment

Again, owing to the differences in perception for each type of recording, reliability of responses was assessed individually for each type of recording. By contrast to the dimensional assessment, the ratings of the Bach10 recordings show a slightly higher agreement than those for Kantorei: AC_1_ = 0.62 for Bach10, AC_1_ = 0.53 for Kantorei. Concerning the perceived emotion in terms of GEMS, clear patterns emerged for both types of recordings. The most frequently perceived factor in the Kantorei recordings was *transcendence* (47.6% of the total; cf. table 4), whereas for Bach10, it was *sadness* and *tenderness* (28.4% each; cf. table 4). This confirms, as expected, that scoring and expressiveness—which differ between the two evaluated types—play a fundamental role in modelling perception of emotions.

**Table 4.** Distribution of the annotators responses (26 annotators × 8 songs) across the emotional factors. (Absolute values (above) and percentage scores (below) for Kantorei and Bach10 recordings are given. Highest scores for each recording type are marked in italics.)

	GEMS factors									
	dysphoria	sadness	sensuality	tenderness	amazement	activation	power	joy	tranquility	transcendence
Kantorei	0	13	1	20	17	1	42	3	12	*99*
%	0.0	6.2	0.5	9.8	8.2	0.5	20.2	1.4	5.8	*47.6*
Bach10	2	*59*	7	*59*	10	0	18	23	21	9
%	0.9	*28.4*	3.4	*28.4*	4.8	0.0	8.6	11.1	10.1	4.3

#### 3.1.3. Discussion

**Figure 4.**
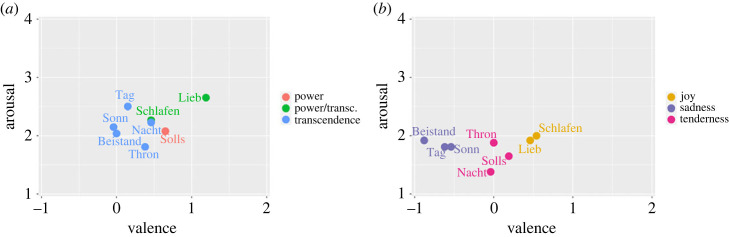
Emotional constellations for the perception of the evaluated chorales. The mean two-dimensional values per choral (*x*: valence; *y*: arousal), already indicated in table 3, as well as the most chosen category, are displayed for each recording type: (*a*) Kantorei and (*b*) Bach10.

Concerning the valence dimension, the perceptual study shows comparable results in the two types of recordings. For both Kantorei and Bach10, listeners associate the chorales Beistand, Sonn and Tag with the left, negative side of the emotional constellation; Thron, Nacht and Solls, with the central area; Lieb with the right, positive side (cf. figure 4). These three areas are clearly defined for the Bach10 recordings, since the associations of chorales across the valence dimension is further confirmed by the categorical ratings: Beistand, Sonn and Tag associated with negative valence and *sadness*; Thron, Nacht and Solls associated with neutral valence and *tenderness*; Lieb and Schlafen associated with positive valence and *joy* (cf. figure 4*b*). This positioning of the emotional categories in the valence dimension goes along with the one already found in the existing literature (cf. fig. 7.3, p. 113, in [1]). Besides the higher condensed constellation for the Kantorei recordings resulting in a lower differentiation of the three areas, another difference to be noticed is that the Kantorei recordings are generally perceived as more positive.

The perceptual results can be interpreted, to some extent, according to music theory principles. Except for Schlafen (in A minor), all the chorales in minor mode show a more negative valence and are clearly associated with *sadness* for Bach10; cf. Beistand, Tag and Sonn in figure 4. The association between the dimensional and categorical ratings for the Bach10 recordings is confirmed by the moderate correlation for valence and the pairwise comparisons that involve *sadness*: *r*_*pb*_ = 0.44 with respect to *tenderness*, *r*_*pb*_ = 0.33 with respect to *joy*; cf. table 5. This indicates that differentiating chorales which express *sadness* from the ones expressing *tenderness* or *joy* might be possible according to the ratings given for valence, a result in line with the typical association between the minor mode and negative emotions [56]. Still, our exploratory results should be corroborated with larger datasets, since indeed, previous works have also shown that perceived negative valence is not necessarily associated with sadness [32].

**Table 5.** Absolute point biserial correlation coefficient *r*_*pb*_ between dimensional ratings (arousal and valence) and the most chosen emotional categories for both type of recordings. (For Kantorei: transcendence (transc.), power/transc. (mixed)^14^ and power; for Bach10: sadness, tenderness and joy. Highlighted in italics *r*_*pb*_ > 0.3. Note that, to compute point biseral correlations, pairwise categories (dichotomously encoded as ‘1’ and ‘0’) should be considered; thus, correlations between the three possible pairwise combinations and each dimension are given for both types of recordings.)

	Kantorei recordings			Bach10 recordings		
	transc.—mixed	transc.—power	power—mixed	sadness—tenderness	sadness—joy	tenderness—joy
arousal	0.14	0.03	0.17	0.05	0.12	0.33
valence	0.30	0.18	0.09	0.44	0.33	0.23

The chorale Schlafen was the only one in minor mode slightly associated with a positive valence. The categorical ratings were to some extent ambiguous. For the Kantorei recordings, the majority of the ratings (69.2%) were equally distributed between *transcendence* and *power*; cf. 34.62% for each in table 6. For the Bach10 recordings, even the category with the highest amount of ratings, *joy*, received a low percentage; cf. 23.08% in table 6. This ambiguity could derive from the harmonic discourse: Schlafen is a chorale in minor mode but all its cadences resolve on major chords (cf. cadences in table 2). Still, compositions in minor mode concluding with major chords were typical before the nineteenth century [7]—a tendency also shown in Sonn, for which no categorical ambiguity is displayed. Our perceptual results suggest that music in minor mode can also be perceived as positive in terms of valence, a ‘mismatched’ association which might lead to perceptual ambiguities when reasoning in terms of categories.

**Table 6.** Frequency distribution (in %) of listeners’ categorical perception of the GEMS emotional factors for both type of recordings: *Katorei* and Bach10. (Higher values are highlighted in italics.)

	GEMS factors									
choral	dysphoria	sadness	sensuality	tenderness	amazement	activation	power	joy	tranquility	transcendence
*Kantorei*										
Solls	0.00	3.85	3.85	7.69	7.69	0.00	*42.31*	0.00	7.69	26.92
Schlafen	0.00	11.54	0.00	3.85	7.69	3.85	*34.62*	0.00	3.85	*34.62*
Tag	0.00	0.00	0.00	15.38	0.00	0.00	7.69	0.00	3.85	*73.08*
Beistand	0.00	7.69	0.00	3.85	3.85	0.00	15.38	0.00	7.69	61.54
Nacht	0.00	0.00	0.00	19.23	7.69	0.00	3.85	0.00	15.38	*53.85*
Sonn	0.00	11.54	0.00	19.23	11.54	0.00	11.54	0.00	0.00	*46.15*
Thron	0.00	15.38	0.00	0.00	11.54	0.00	15.38	0.00	3.85	*53.85*
Lieb	0.00	0.00	0.00	7.69	15.38	0.00	*30.77*	11.54	3.85	*30.77*
*Bach10*										
Solls	0.00	19.23	7.69	*30.77*	0.00	0.00	7.69	19.23	15.38	0.00
Schlafen	0.00	15.38	0.00	11.54	15.38	0.00	7.69	*23.08*	15.38	11.54
Tag	0.00	*46.15*	0.00	34.62	0.00	0.00	11.54	0.00	7.69	0.00
Beistand	0.00	57.69	7.69	23.08	3.85	0.00	0.00	0.00	0.00	7.69
Nacht	0.00	23.08	0.00	*46.15*	7.69	0.00	7.69	7.69	7.69	0.00
Sonn	3.85	*42.31*	0.00	23.08	3.85	0.00	15.38	0.00	7.69	3.85
Thron	0.00	19.23	7.69	*34.62*	3.85	0.00	11.54	7.69	11.54	3.85
Lieb	3.85	3.85	3.85	23.08	3.85	0.00	7.69	*30.77*	15.38	7.69

From a categorical point of view, Solls was the only chorale clearly related to *power* in the Kantorei recordings. This might be explained by the fact that Solls is also the most harmonically stable chorale, as shown by cadences resolving only in the tonic and in the dominant; cf. I (tonic) and V (dominant) cadences in table 2. However, this connection between harmonic stability and *power* is not shown for the Bach10 recordings, where this chorale was perceived as related to *tenderness*. This result suggests that besides harmonic stability, the expressiveness of the choir played an important role in conveying *power*; this is in line with previous works which highlight the ability of the singing voice in communicating emotions [28]. Similarly, listeners show a clear tendency towards choosing *transcendence* for the Kantorei recordings (cf. five chorales in blue in figure 4*a*); this could be expected as this factor is represented by adjective markers highly related to religious concepts, such as ‘mystic’ or ‘spiritual’. However again, none of the chorales from the Bach10 recordings was identified with this category, which confirms the importance of scoring.

From our exploratory results we can observe that the perception of the Kantorei and the Bach10 recordings is comparable for valence, but clearly distinct for emotional categories. This suggests that expressiveness and scoring played a more prominent role in our listeners’ perception of emotional categories. Concerning valence, our results—even if based on a very small sample—are in line with previous works suggesting that the perception of valence is more affected by compositional cues such as mode than expressive ones [57,58]. However, owing to the restricted range of the scores obtained for arousal, no clear evaluation of this dimension can be performed in our study. This might indicate that despite its religious nature, the evaluated repertoire does not sufficiently convey differences in the perceived arousal. After evaluating our results, we hypothesize that investigating sacred music with an even more clearly defined meaning within the liturgy, such as Mass’ prayers, might be needed in order to gain a better understanding of the arousal dimension.

### 3.2. Data-driven study

In this section, we address RQ2: *are there any relationships between perception of emotion and machine-based features?* We examine the association between perceived emotions and the features extracted from the three investigated modalities: lyrics, codified scores and audio recordings. As emotional lexica and word embeddings enabled us to map the lyrics of the chorales onto emotional dimensions and GEMS factors (cf. §2.2.1), following the structure of the perceptual analysis (cf. §3.1), the dimensional and GEMS assessments of the linguistic features will be presented individually, followed by a general discussion. Subsequently, in order to detect potential associations between perceived emotions and symbolic (musical) and acoustic features of the chorales, exploratory factor analysis (EFA) will be employed. Results from machine-based features will be compared with the outcomes obtained from listeners’ dimensional ratings. Through EFA we aim to uncover commonalities between the investigated chorales by modelling latent factors (extracted through maximum likelihood) of the chorales’ features vectors. EFA enables us to assess how individual chorales contribute to explaining the variance of such factors. This type of evaluation is the most suitable for our small dataset, for which supervised ML methods, such as classification or regression, are not appropriate. Note that despite the small number of evaluated samples, in order to avoid biases and guarantee the generalizability of the experiments, before performing the EFA, the suitability of the selected features (previously retrieved from the whole dataset) will be assessed for the small sample.

#### 3.2.1. Linguistic features

##### 3.2.1.1. Linguistic features: dimensional assessment

**Figure 5.**
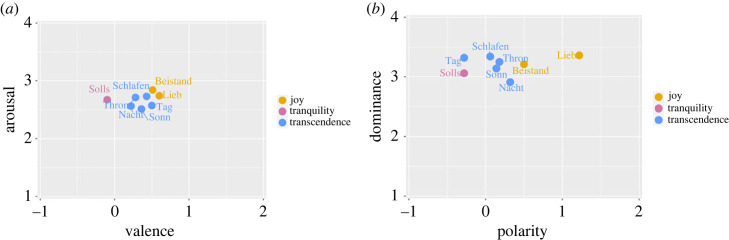
Emotional constellations extracted from the lyrics. The two-dimensional mappings computed from the mean scores (*x*: valence; *y*: arousal) and (*x*: polarity; *y*: dominance) are shown in (*a*,*b*), respectively. The factors showing a higher proximity to the lyrics are also indicated for each evaluated chorale.

Mean valence and polarity retrieved from the lyrics are generally similar for all chorales. Except for Tag that displays a positive valence (0.5 in figure 5*a*) and a negative polarity (−0.28 in figure 5*b*), the other chorales are similarly distributed over both dimensions: Solls on the left (more negative) side of the constellation (−0.10 for valence, −0.28 for polarity); Schlafen, Thron, Sonn, Nacht in the central (neutral) area; Beistand (to some extent) but especially Lieb towards the right (more positive) side (0.60 for valence, 1.22 for polarity); cf. figure 5*a*,*b*. This is not surprising, as valence and polarity both refer to the emotional hedonic value. The mean scores for arousal and dominance show a much more restricted range, that is, the difference between the highest and the lowest scores is considerably lower than the one displayed for valence and polarity: arousal range =0.33 and dominance range = 0.45; valence range = 0.70 and polarity range = 1.50. The restricted range for arousal and dominance becomes obvious when considering the value of the *y*-axis in figure 5*a*,*b*. This contrasts with the larger range for valence but specially for polarity, which is visually shown by the spread of emotional factors over the *x*-axis of the plot (cf. polarity in figure 5*b*).

Our assessment shows that the perception of emotional dimensions can be partially associated with the dimensional values extracted from the lyrics. The valence scores extracted from the lyrics and those obtained from the perception of the Kantorei recordings show that the chorale with the most positive valence was Lieb for both listeners and lyrics: 1.19 and 0.60, respectively (cf. Lieb in figures 4*a* and 5*a*). By contrast, the one with the most negative valence according to the lyrics was Solls (cf. −0.10 in figure 5*a*), while from the listeners perspective, it was Sonn (cf. −0.04 in figure 4*a*). This relationship was supported by a positive correlation between the valence perceived from the Kantorei recordings and the polarity extracted from the lyrics (*Pearson*’s *r* = 0.5). Except for a moderate correlation between the arousal perceived from the Bach10 recordings and the one retrieved from the lyrics (*r* = 0.5), all the other correlations yielded results −0.2 ≤ *r* ≤ 0.2. However, owing to the restricted range of scores for arousal, we do not consider this correlation to be particularly meaningful.

When looking at the lyrics, the association of Lieb with a positive valence becomes obvious, as this chorale, besides including positive terms such as ‘sweet’ or ‘peace’, mentions several times the word ‘love’.^15^ Similarly, the association of Solls with negative valence is also immediate when looking at the lyrics, clearly related to negative terms such as ‘punishment’, ‘pain’, ‘sin’ and ‘penance’.^16^ Since the listeners did not evaluate the lyrics explicitly but the performance as a whole, the fact that Solls was not associated by the listeners with a negative valence for any of the recordings suggests that the negative valence of the lyrics is not supported by musical/expressive cues encoded in the performance, which we expect to have a stronger impact on listeners’ perception.

##### 3.2.1.2. Linguistic features: Geneva Emotion Music Scale assessment

The similarities between the lyrics of the chorales and the GEMS factors are given in table 7. A 1 indicates maximal similarity, −1 maximal dissimilarity.

**Table 7.** Mean cosine similarity between the chorales and the GEMS emotional factors according to the lyrics. (For each chorale, the highest similarity is marked in italics. Values in italics face indicate the highest absolute value for each choral.)

	dysphoria	sadness	sensuality	tenderness	amazement	activation	power	joy	tranquility	transcendence
Solls	−0.10	0.06	−0.06	0.04	0.02	−0.02	0.03	0.05	*0.16*	0.09
Schlafen	−0.13	−0.06	0.02	0.05	0.10	0.07	0.14	0.16	0.16	*0.21*
Tag	−0.14	0.00	0.00	0.10	0.08	0.03	0.13	0.17	0.20	*0.29*
Beistand	−0.21	−0.17	0.05	0.07	0.16	0.09	0.23	*0.26*	0.16	0.26
Nacht	−0.10	−0.04	0.02	0.08	0.09	0.07	0.16	0.18	0.16	*0.25*
Sonn	−0.15	−0.02	0.00	0.08	0.06	0.03	0.10	0.12	0.19	*0.23*
Thron	−0.14	−0.05	0.00	0.07	0.09	0.04	0.14	0.17	0.20	*0.22*
Lieb	−0.21	−0.18	0.09	0.13	0.18	0.09	0.22	*0.29*	0.22	0.26

*Sensuality* is clearly unrelated to all the chorales. This can be explained by the religious nature of the evaluated music and is shown by all the scores being close to 0; cf. sensuality in table 7. The factors with a negative valence—*sadness* but especially *dysphoria*—are dissimilar to the chorales, as shown by the predominance of negative scores for all the chorales; cf. dysphoria and sadness in table 7. This suggests that the emotional content expressed by the lyrics is positive, which is confirmed by the fact that most of the chorales clearly relate to *transcendence* and to some extent to *joy*, *tranquility* and *power*, as shown by the relatively high scores for these categories; cf. italic values in table 7.

Both the listeners’ ratings of the Kantorei recordings and the emotional categories extracted from the lyrics show a predominant association of the chorales with the factor *transcendence* (cf. Tag, Beistand, Nacht, Sonn and Thron in table 7 and in figure 4*a*). This is supported by correlation results between the cosine similarity computed on the machine-based features and the frequency distribution of the perception ratings of each chorale across factors. Following this approach, high positive correlations would indicate that the same associations between a chorale and the GEMS factors is displayed for both machine-based features and perception results. In particular, the chorales Tag and Nacht yielded *r* > 0.6 (cf. Kantorei in table 8). Similarly, the chorale Lieb is clearly associated with *joy* by both listeners (cf. figure 4*b*) and the lyrics (cf. 0.29 in table 7), which is to some extent supported by a moderate correlation (cf. *r* = 0.49 for Bach10 in table 8).

**Table 8.** Results of Pearson correlation computed between the machine-based cosine similarity vectors and the frequency distribution of perceptual ratings for each chorale. (Correlations between machine-based ratings and perception are given considering the perceptual ratings of both the Kantorei and the Bach10 recordings, separately.)

	chorale							
	Solls	Schlafen	Tag	Beistand	Nacht	Sonn	Thron	Lieb
Kantorei	0.29	0.47	0.64	0.41	0.62	0.52	0.44	0.62
Bach10	0.40	0.48	−0.11	−0.52	0.10	−0.14	0.04	0.49

Differently, the association between the lyrics of the chorale Beistand and *joy* is clearly contradicted by the listeners’ perception of the Bach10 recordings, where this chorale is the one perceived as most negative in terms of valence and associated with *sadness* (cf. figure 4*b* and table 7); again, this is supported by a negative correlation (cf. *r* = −0.52 for Bach10 in table 8).

##### 3.2.1.3. Linguistic features: discussion

From our experimental results, associations between perception and the emotions mapped onto the lyrics can be drawn. Yet, listeners’ ratings—which we assume are more influenced by the musical cues—might not necessarily coincide with the emotional meaning of the lyrics. The divergences between perceived emotions and the ones extracted from the lyrics become evident for the chorale Solls: associated with *tranquility* according to the lyrics but with *power* and *tenderness* according to the perception of the Kantorei and Bach10 recordings, respectively. This confirms that the association of this chorale with *power* is owing to the choir’s expressiveness [28], which cannot be captured either from the lyrics, nor from the Bach10 recordings.

The perceptual results for Beistand—a chorale in minor mode clearly associated by the listeners with negative valence and *sadness* but related to positive valence and *joy* according to the lyrics—show that the musical cues are of higher importance for listeners than the lyrical ones. This is in line with previous research [23] showing that, with respect to a target emotion, listeners’ examination of congruent melodies and mismatched lyrics yields higher agreement that congruent lyrics with mismatched melodies. This would align with the general belief that minor tonalities are associated with perceived negative emotions, a musical cue that seems to have a higher influence on perception than mismatched lyrical content.

Confirming the results obtained from the perceptual study, no meaningful conclusions can be drawn from the arousal and dominance scores retrieved from the lyrics, as they all lay in a very reduced range of values (cf. figure 5). This confirms previous work [59], which showed that lyrics may be a more suitable source to encode emotional dimensions related to the hedonic value (i.e. valence) rather than those related to the intensity and control (i.e. arousal and dominance, respectively). This is also in line with speech and emotion research, where it is well established that valence is much more encoded in linguistics and arousal much more in acoustics [60].

Finally, it is important to mention that the moderate (instead of high) correlation between perception and machine-based results for the chorale Lieb (cf. *r* = 0.49 Bach10 in table 8) is owing to the high similarity ratings shown between this chorale and other emotions besides *joy* (in particular *trascendence*, cf. 0.26 in table 7). This indicates that the metrics typically used to annotate MER datasets, such as annotators’ majority vote or higher similarity in terms of words embeddings, might hinder more complex patterns to emerge; thus, distributions of ratings should be taken into account when assessing emotion categories.

#### 3.2.2. Symbolic features

When evaluating the loadings between the selected features and the PCs extracted from the eight chorales, the results are comparable to those shown for the Bach300+ symbolic dataset containing 362 chorales (cf. figure 2). For both, the features with a higher correlation (both positive and negative) with the first PC are *pitch class kurtosis after folding* and *pitch class variability after folding*, displaying a positive and negative coefficient, respectively: 0.41 and −0.39 for the Bach300+ dataset (cf. figure 2); 0.44 and −0.43 for the eight evaluated chorales. This suggests that the selected features are robust enough to efficiently capture the main characteristics of the evaluated repertoire, not only for the large dataset (362 chorales out of 370), but also for the small set, i.e. the remaining eight chorales, evaluated in detail in our study.

**Figure 6.**
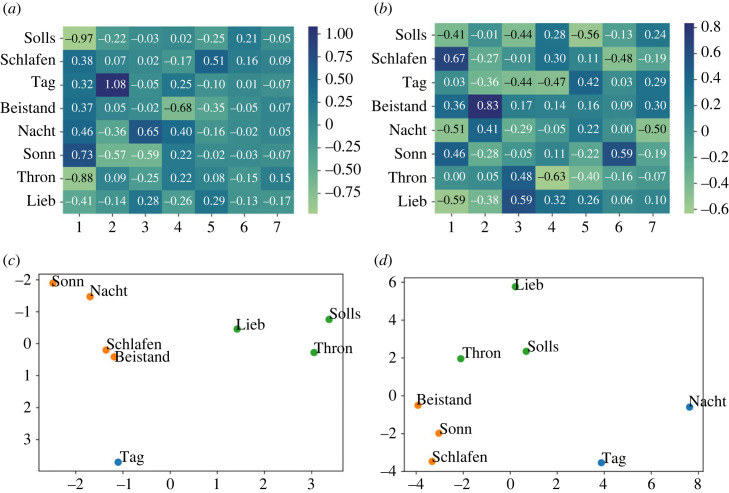
Factor loadings and *k*-means clustering visualized according to the two principal components (*x*: PC1; *y*: PC2) for the eight evaluated chorales. Comparative results computed from the symbolic features and from the listeners’ ratings are displayed. (*a*) Factor loadings—symbolic, (*b*) factor loadings—perception, (*c*) clustering—symbolic and (*d*) clustering—perception.

In figure 6, factor loadings from the EFA are displayed for the machine-based symbolic features in comparison to the perceptual results. For visualization purposes, *k*-means clustering with *k* = 3 (chosen as optimal number based on the ‘elbow’ according to the average distance to cluster centroids) are also displayed. The chorales belonging to each cluster are identified with the same colour, which is randomly assigned. Note that the two-dimensional plots are based on the two PCs explaining most of the variability in the data. Since the symbolic representation is the same for both recordings, ratings for both the Kantorei and Bach10 recordings, as well as both arousal and valence dimensions, were aggregated before computing the EFA for perception. Perceptual ratings of valence and arousal were already individually assessed in §3.1; therefore, we now intentionally integrate the ratings from both dimensions in order to achieve a more general representation of the perceived emotion that can be directly compared with the machine-computed features.

The EFA based on the symbolic features shows a strong negative relationship between the factor 1 and the chorales Solls, Thron and to some extent Lieb, as this factor explains most of the variance represented in the symbolic features extracted from these chorales (cf. coefficients −0.97, −0.88 and −0.41, respectively, in figure 6*a*). This factor shows positive coefficients for all the other chorales. This can be consequently observed in the clusters representing the chorales through *k*-means, where the three chorales belong to the green cluster (see right side of the constellation), while the others belong to other clusters (see the left side); cf. figure 6*c*. The distinction of these chorales in the green cluster with respect to the other chorales can be seen for perception too (cf. figure 6*d*).

**Figure 7.**
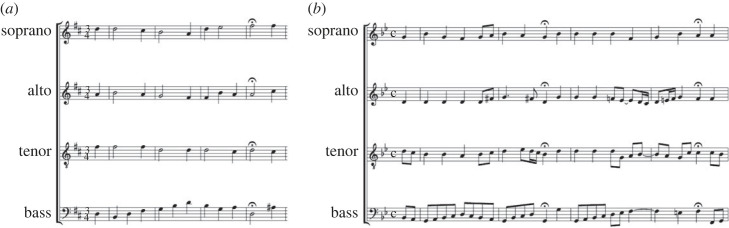
First four bars of the chorales (*a*) Thron and (*b*) Tag.

The distinction of the chorales included in the green cluster can be interpreted from a musical point of view. On the one side, the three chorales are in Major mode and present predominantly stable cadences (cf. most of the cadences for these chorales in either I or V; see table 2). On the other side, the three chorales are characterized by the use of relatively long rhythms (quaver notes are the shortest one), something evident for Thron, containing almost only minims and crochet notes. This restricted rhythmic variety contrast with the rest of evaluated chorales, in particular Tag (the only one displayed in the blue cluster), as can be seen when comparing the first bars of both chorales (cf. figure 7*a*,*b*): both distant to each other for the symbolic and perceptual *k*-means results (cf. figure 6*c* and 6*b*).

Finally, by examining the factor loadings for the symbolic features, some patterns already described for perception can be identified. On the one side, our earlier observation that Solls was perceived as distinct in terms of emotion categories (clearly identified with the category *power*) is supported by the fact that this chorale is highly explained by the factor 1 (cf. −0.97 in figure 6*a*). On the other side, an ambiguous chorale such as Schlafen (ratings equally distributed between *transcendence* and *power*), cannot be explained by the EFA, which is shown by the lowest range of factor loadings, i.e. 68 (cf. −0.17 ≤ coefficients ≤0.51 for Schlafen in figure 6*a*).

#### 3.2.3. Acoustic features

For both types of recordings, the loadings between the selected acoustic features and the PCs extracted from the eight evaluated chorales are similar to those obtained from the Bach300+ audio dataset. For the Kantorei recordings—as already shown for the Bach300+ audio dataset, the features with a higher positive correlation with the first PC were those related to the fourth MFCC except for skewness: for Kantorei ≥0.32; for the Bach300+ audio dataset ≥0.25 (cf. figure 3). The opposite trend is displayed for the Bach10 recordings, for which the acoustic features related to the fourth MFCC (except for skewness) show the strongest negative correlations: less than or equal to −0.4. This difference might be owing to the diversity in terms of instrumentation between both type of recordings: while the recordings from the Bach300+ audio dataset, as well as the Kantorei ones, are performed by a choir, those by Bach10 are not. Still, the fact that the fourth MFCC presents a negative correlation with the first PC might not necessarily indicate that the features are irrelevant. On the contrary, since the absolute values are similar, for the Bach10 recordings, the selected features might be just discriminative in the opposite direction.

**Figure 8.**
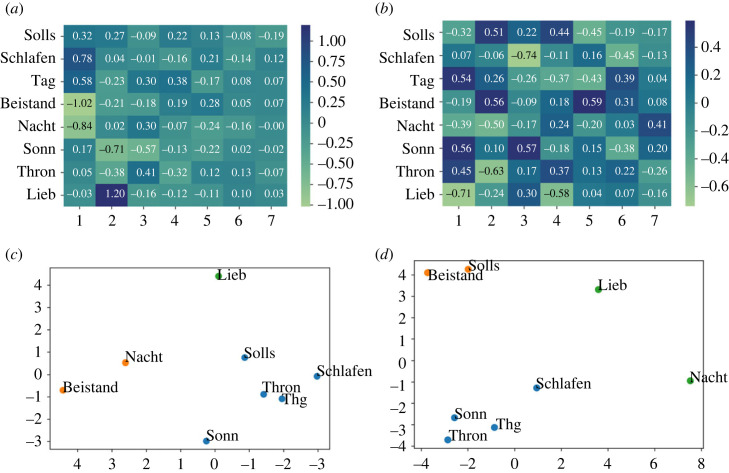
Factor loadings and *k*-means clustering visualized according to the two principal components (*x*: PC1; *y*: PC2) for the Kantorei recordings of the eight evaluated chorales. Comparative results computed from the acoustic features and from the listeners ratings are displayed. (*a*) Factor loadings—Kantorei, (*b*) factor loadings—perception, (*c*) clustering—Kantorei and (*d*) clustering—perception.

In figure 8, factor loadings from the EFA are displayed for the acoustic features extracted from the Kantorei recordings in comparison to the perceptual results for the same recordings. Again, to visualize the results, two-dimensional plots showing *k*-means outcomes with *k* = 3 are shown. As already described for the EFA based on the symbolic features, for the perceptual assessment of the Kantorei recordings, both arousal and valence dimensions were aggregated before computing the EFA for perception, by this obtaining a general representation of the perceived emotion.

The EFA for the Kantorei recordings displays a prominent dissimilarity pattern for both perception and acoustics, i.e. the chorale Beistand located in the opposite position with respect to chorales from the blue cluster. For the acoustic features, this is clear for Beistand versus Schlafen (cf. −1.02 versus 0.78, factor 1 in figure 8*a*); for the perceptual features this is shown for Beistand versus Thron (cf. 0.56 versus −0.63, factor 2 in figure 8*b*). Musically, this might be owing to rhythmic differences: Thron containing almost only minims and crochet notes (cf. figure 7*a*); Schlafen showing a prevalence of quaver notes; Beistand, characterized by a rhythmic bass contrasting with the upper parts, in between.

Interestingly, unlike for the Kantorei recordings, the distinction between the three chorales was clear for the Bach10 recordings, where each chorale was associated with a different emotional category (cf. figure 4*b*). The fact that categorical perception showed a higher inter-rater agreement (AC_1_) for the Bach10 recordings than for the Kantorei ones (cf. §3.1.2), seems to support the idea that a more ‘real’ recording, i.e. performed by a choir, could automatically evoke in the listeners the emotion *transcendence*, by this producing an occlusion effect which would impair listeners’ discrimination capabilities in terms of emotion categories. On the contrary, a recording without such a sonority and less expressive, might evoke more clearly differences in emotion categories which can be related to the musical content. This was, however, only partially supported by the symbolic features, which—although clearly capturing the distinctiveness of Thron—failed to differentiate between Beistand and Schlafen.

**Figure 9.**
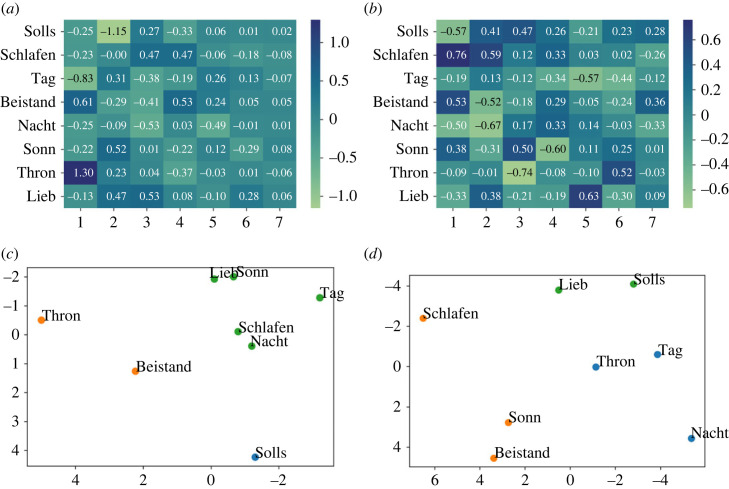
Factor loadings and *k*-means clustering visualized according to the two principal components (*x*: PC1; *y*: PC2) for the Bach10 recordings of the eight evaluated chorales. Comparative results computed from the acoustic features and from the listeners ratings are displayed. (*a*) Factor loadings—Bach10, (*b*) factor loadings—perception, (*c*) clustering—Bach10 and (*d*) clustering—perception.

Finally, the EFA for the Bach10 recordings does not present patterns clearly shared between the acoustic and the perceptual representations. We hypothesize that this might be owing, on the one side, to the selected features which, as already mentioned, might not totally capture the most salient information for the Bach10 recordings so well as for the Kantorei ones.^17^ This would be confirmed by the colour shadowing from figure 9*a*, mostly displaying a shadowing around the medium values. On the other side, annotators’ disagreement might have lead to contradictory information which can be seen when looking at the factor loadings for perception: for instance, Schlafen versus Beistand (cf. figure 9*d*), showing high positive coefficients for factor 1 (0.76 versus 0.53) but displaying opposite magnitudes for factor 2 (0.59 versus −0.52). Such a complexity results in larger distances between chorales in the two-dimensional space, which—although belonging to the same cluster—often fall apart from each other. Since this spread is not shown for the two-dimensional visualization obtained from the perception of the Kantorei recordings, this seems indeed to be owing to the lower ICC obtained in the perception of arousal for the Bach10 recordings with respect to the Kantorei ones (cf. §3.1.1).

## 4. A multi-modal exploratory assessment based on unsupervised machine learning

**Figure 10.**
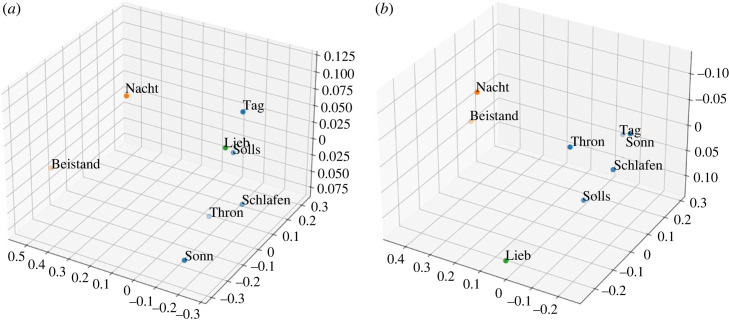
Multi-modal clusters of chorales for the most informative combinations of the evaluated features computed through *k*-means and visualized according to the three principal components (*x*: PC1; *y*: PC2; *z*: PC3). In (*a*) two-modal clusters computed from linguistic and acoustic features extracted from the Kantorei recordings are visualized; in (*b*) three-modal clusters computed from linguistic, symbolic and acoustic features extracted from the Kantorei recordings are shown.

In this section, we assess RQ3: *do connections between the emotional characterizations of the music as obtained from perception and as generated from unsupervised ML techniques based on multi-modal features exist?* We carry out a more integrative comparison across the investigated features by performing multi-modal clustering again with *k*-means (*k* = 3). The clustering was carried out for each of the three modalities individually as well as in all possible combinations. For the linguistic features, the eight-dimensional vectors containing the mean and standard deviation across the emotional dimensions were considered. For the symbolic and acoustic ones, the selected features (vectors of 12-dimensions and 14-dimensions, respectively), extracted from the MIDI files as well as from the Kantorei and the Bach10 recordings, were used. As in the previous experiments, we visualize the clusters using the projection of the features onto the top PCs, now three in order to generate three-dimensional visualizations (cf. figure 10). Owing to the larger dimensionality of the feature representations, the results are now visualized through three-dimensional plots, which are, in this case, more informative.

The *k*-means results show that the linguistic features extracted from the lyrics are the modality for which less variance in the data can be explained by the clustering (61.6%) which is below the 71.3% explained by the symbolic features as well as by the 76.9% and 69.5% explained by the acoustic features extracted from the Kantorei and the Bach10 recordings, respectively. When combining two modalities, the explained amount of variance is comparable for the combinations involving lyrics: 70.5%, 76.7% and 69.0% explained by the combination of lyrics with symbolic, Kantorei and Bach10 features, respectively. Differently, the percentage of total variance in the data that can be explained by the *k*-means clustering decreases for the combinations among symbolic and acoustic features: 65.6% and 61.1% explained by the combination of symbolic with Kantorei and Bach10 features, respectively. As expected, the combination between the two types of acoustic features results in a decreased explained variance as well, i.e. 64.1%. This is owing to the fact that symbolic and acoustic features may contain redundant information, negatively impacting the clustering. The amount of variance explained by the combination of the three modalities decreased even further for the combination involving the Bach10 recordings (i.e. 60.9% for lyrics, symbolic and Bach10), while it remained constant for the one involving the choir recordings (i.e. 65.6% for lyrics, symbolic and Kantorei). To sum up, the multi-modal solutions for which a higher variance is explained by the *k*-means clustering are those involving the acoustic features extracted from Kantorei recordings, i.e. the two-modal solution combining linguistics and Kantorei (76.7% variance explained) and the three-modal solution combining linguistics, symbolic and Kantorei (65.6% variance explained). Therefore, in the following, only the results from these will be interpreted.

**Figure 11.**
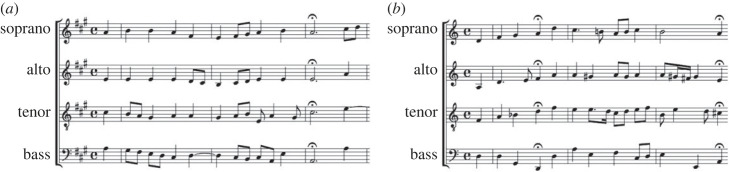
First three bars of the chorales (*a*) Lieb and (*b*) Sonn.

In figure 10, the clustering results show that the distribution of chorales among clusters is the same for both the two-modal and the three-modal solutions. This is indeed the same distribution already displayed by the mono-modal results extracted from the Kantorei recordings (cf. figure 8*c*), which indicates the prominent role played by the acoustic features in the displayed results. Nevertheless, when comparing the two-modal with the three-modal solutions, the distance between Lieb with respect to Sonn is more pronounced in the latter. This indicates that the addition of symbolic features enables a better distinction between both chorales, which indeed were also clearly distinct from the symbolic evaluation (cf. figure 6*c*); these differences can be related to textural musical properties: Lieb characterized by softer melodic contours and musical motives often based on repeated notes as well as parallel diatonic motion (cf. in particular the two higher voice parts in figure 11*a*); Sonn showing a slightly more chromatic and syncopated contour based also on the use of larger intervals (cf. figure 11*b*). In addition, Lieb is displayed in a clear distinct position with respect to the other chorales. This contrasts with the two-modal solution, where the chorale Beistand (to some extent ambiguous since clearly associated with positive lyrics but in minor mode) was the most distinct.

These results can be related to the outcomes from the listeners perception, as Lieb and Sonn are displayed in the extreme opposite position of the dimensional constellation in terms of valence and Lieb is also clearly represented as distinct with respect to the others (cf. figure 4*a*). The obtained results suggest that modelling a higher amount of modalities enables us to more efficiently capture complex perception patterns, especially when contradictory messages might arise from different feature modalities.

## 5. Conclusion

We now outline the results of our study as well as their limitations, detailed for each research question. A summary of the key conclusions is given in table 9.

**Table 9.** Summary of the key results obtained from the presented work.

study	conclusions
user-based	(i) as Kantorei recordings (unlike Bach10) are clearly associated by listeners to *transcendence*, choir sonority seems to be essential in transmitting the mystic connotations of sacred music
	(ii) the perception of valence is similar for the Kantorei and Bach10 recordings, while the perception of emotional categories differs; we assume that expressiveness and scoring (different for both types of recording) impact more prominently the perception of music emotions in terms of categories than in terms of dimensions
	(iii) although the minor mode is generally associated by listeners with negative valence, its association with the positive one generates ambiguity in the perception of emotional categories
data-driven	
*linguistics*	(i) lyrics seem to be a better source for encoding valence than arousal and dominance
	(ii) the association between positive lyrics to minor mode shows that the linguistic content might contradict the musical cues; perception seems to be more influenced by the latter
*symbolics*	(i) the distinctiveness/ambiguity of perceived emotional categories can be identified from symbolic features through exploratory factor analysis
	(ii) the ability of choir sonority to inspire the emotion *transcendence* might produce an occlusion effect which impairs listeners’ discrimination capabilities in terms of fine-grained categories; yet, such categories can be to some extent distinguished from symbolic features
*acoustics*	(i) through acoustic features, it is possible to mirror dissimilarity patterns observed in human perception of emotional dimensions
	(ii) the role of expressiveness and scoring in influencing perception of music emotions can be—to some extent—inferred from the acoustic features extracted from the recordings
ML-based	(i) when combining features extracted from several modalities, the amount of variance in the data that can be explained by ML methods, such as clustering, stays constant for highly dissimilar modalities, e.g. lyrics and acoustics, but decreases for multi-modal representations that might contain redundant information, e.g. symbolics and acoustics
	(ii) modelling multi-modal features is useful in mirroring perception patterns, especially when contradictory messages arise from different modalities (e.g. positive lyrics and minor mode)

RQ1. *To which extent can the perception of emotion in the selected chorales be related to their musical properties?*

By comparing perception of emotions in two types of recordings, we have shown that scoring and expressiveness are essential properties to evoke the mystic feelings underlying sacred music; cf. (i) for user-based study in table 9. This is in line with many works highlighting the role of performances in evoking specific emotions in listeners [61]. Indeed, the Kantorei recordings, characterized by a particular scoring typical of sacred music (choir) and showing expressive professional interpretations, were mostly related to the emotional factor *transcendence*, with obvious religious connotations. This might also be related to the emotion regulation effects typically attributed to singing in a choir [62], a type of sonority that might have an impact on the listeners as well.

Our work also shows that the differences in perception between the evaluated recordings become much more prominent for the categorical than for the dimensional assessment; cf. (ii) for user-based study in table 9. This suggests that expressiveness and scoring might play a more prominent role when reasoning in terms of emotional categories, which highlights the importance of carefully selecting the recordings when evaluating perception of emotions. The importance of using historically informed performances has already been pointed out by Bowan [63].

The perceptual results generally confirm the typical belief that minor tonalities relate to negative valence [64] and to emotions such as sadness, at least in Western culture [65]. Still, the relationship between mode and valence is an object of controversy owing to contradictory outcomes shown in previous works [32,56]. This is corroborated by the fact that the exceptions, that is, the musical examples in minor tonalities associated with positive valence, yield emotional ambiguities (high listeners’ disagreement) when reasoning in terms of categories; cf. (iii) for user-based study in table 9. On the one side, this is consistent with previous works indicating the poorer resolution of categorical models in characterizing ambiguous samples [32]. On the other side, this might suggest that employing a categorical model to investigate emotions in music [33] will also be of limited use for domain-specific categorical models.

RQ2. *Are there any relationships between perception of emotion and machine-based features?*

The results from the data-driven study show that—unlike arousal and dominance—the valence dimension is clearly encoded in lyrics; cf. (i) for linguistic in table 9. This was already pointed out in previous work [59]. However, our experimental results show that concepts expressed by the lyrics might not necessarily be confirmed by the musical cues. In such an ambiguous situation, perception was more influenced by the latter, as shown by the general perception of negative valence for chorales in minor mode; cf. (ii) for linguistic in table 9.

Our results also show that ambiguities, as well as distinctiveness, in perceived emotional categories can be identified through EFA, as shown by the symbolic features, whose factor loadings for ambiguous chorales presented a reduced range, while for distinct chorales, it presented the highest absolute values; cf. (i) for symbolics in table 9. Our EFA also indicates that fine-grained categories, whose identification is compromised for perception when listening to ‘real’ performance (in terms of expressivity and scoring), might be to some extent distinguished from symbolic features; cf. (ii) for symbolics in table 9.

Our data-driven study indicates that through acoustic features, it is possible to mirror dissimilarity patterns observed in human perception; cf. (i) for acoustics in table 9. In addition, it further confirms the importance of considering an appropriate performance, as expressiveness and scoring, both relevant for the perception of music emotions, can also be inferred from the acoustic features extracted from the recordings; cf. (ii) for acoustics in table 9.

RQ3. *Do connections between the emotional characterizations of the music as perceived by listeners and as generated from ML techniques based on multi-modal features exist?*

The results obtained from the cluster analysis indicates that, when different modalities are combined, the amount of variance in the data that can be explained by the clustering stays constant for highly dissimilar modalities, e.g. lyrics and acoustics, but decreases for multi-modal representations that might contain redundant information, e.g. symbolics and acoustics; cf. (i) for the ML-based study in table 9. Our analysis shows that a multi-modal integrative approach can be particularly useful when ‘contradictory’ information might be encoded by different feature modalities, such as positive lyrics put in music through minor tonalities; cf. (ii) for the ML-based study in table 9.

Despite the potential of assessing Bach’s chorales for a multi-modal study of emotions in music, it has been pointed out that the very nature of a four-part chorale style might impair the use of affective-symbolic musical figures typically used by Bach to enhance text-tone relationship [66]. This might explain the contradictory information derived from the lyrics and the musical modalities. Thus, in the future we also plan to explore works from other composers as well as a musical religious repertoire showing more defined emotional connotations, such as Mass’ prayers. In addition, although the religious repertoire is expected to be emotionally more homogeneous than others, segmenting prayers into their individual verses might be an option to consider in order to better understand internal textual and musical contrast.

The multi-modal approach, which in contrast to a single-modal investigation seems to be more suitable for mirroring perception, is one of the most distinctive features of our study. Still, despite performing a feature selection strategy based on more than 300 chorales, the generalisability of the presented results is impaired by the small size of the evaluated sample (i.e. eight chorales). Yet, we consider that performing an exploratory study with a reduced dataset was necessary at this stage of our research, where we aimed, for the first time, to apply a transdisciplinary methodology. On the one side, a small dataset allowed us to perform a detailed one-to-one comparison between listeners’ perception of both types of recordings according to two emotional models of emotion. On the other side, it enabled us to carry out an integrative assessment of the results from a perceptual, musicological and computational point of view—note that all the data-driven results are interpreted according to listeners’ perception, which is only available for eight chorales. We believe this integrative assessment is essential in a transdisciplinary method; it would have been very difficult for a larger dataset.

Despite the mentioned limitations, we would also like to highlight that the transdisciplinary methodology herein presented will make it possible to develop new connections among the disciplines involved in music and emotion research. This is important, as such a connection is still missing in the current state-of-the-art. We expect our work to serve as an example of how transdisciplinary knowledge might enable a holistic understanding of emotions in music, by this opening new research horizons towards a more integrative and comprehensive vision of the interplay between computer science, musicology and psychology.

## Data Availability

For reproducibility, we make the code and data freely accessible (doi:10.5281/zenodo.10053401) [67].

## Footnotes

1 https://github.com/juansgomez87/datasets_emotion.

2 Note that with the term ‘codified’, we refer to machine readable symbolic representations. Unlike ‘digitized’ sources (i.e. image-based) in which music and historical texts are typically preserved, codified sources enable the automatic processing of the content by computational means.

3 https://zenodo.org/records/10053402.

4 Note that in the codified music notation, that is, the MIDI files, information on instruments is disregarded because it is irrelevant—the symbolic representation is the same irrespective of the instrument performing it.

5 All the chorales were performed by the Gaechinger Kantorei conducted by Helmuth Rilling (Haenssler CLASSIC).

6 https://verovio.humdrum.org/.

7 https://www.bach-cantatas.com/Texts/IndexTexts7.htm.

8 In the Bach10 dataset B01 is referred to as *Ach Gott und Herr*, B02 as *Ach Lieben Christen*, B10 as *Nun Bitten*.

9 Note that these might differ with respect to the felt emotion, that is, those induced in the listener.

10 Take these lines from B02: ‘Through Adam death comes to us/Christ helps us in all our troubles’. We must not summarize them as a ‘positive’ message because Christ helps us in the end, the same way as we summarize a film review as negative or positive. The world is a vale of tears (death, troubles) but we hope for Christ—the message is both strongly negative and strongly positive, conveyed by the emotional connotations of the words.

11 www.nltk.org/.modules/nltk/sentiment/vader.html.

12 The three adjectives per factor are the following: tenderness (dreamy, nostalgic, melancholic); amazement (amazed, admiring, fascinated); tranquility (calm, meditative, serene); joy (joyful, happy, radiant); activation (exited, active, agitated); power (triumphant, proud, heroic); sensuality (sensual, desirous, aroused); transcendence (spiritual, mystic, illuminated); dysphoria (anxious, angry, frightened) and sadness (sorrowful, depressed, sad).

13 https://github.com/craigsapp/bach-370-chorales.

14 The chorales in the ‘mixed’ category received the same amount of perceptual ratings for both emotional factors, that is, transcendence and power (cf. Schlafen and Lieb in table 3).

15 The translated lyrics for Lieb are as following: *You sweet love, grant us your flavour; Let us feel your burning love; So that we may love each other from the heart; And in peace remain of one mind; Lord, have mercy.*

16 The translated lyrics for Solls are as follows: *If it indeed must be; That punishment and pain; Must follow sin; Then here continue; And there take care of me; And here let me do penance.*

17 Note that, in order to promote the generalisability of our experimental results, the feature selection was performed on the Bach300+ audio dataset, which—unlike the Bach10 recordings—contained choir performances.
